# Association between Presenteeism, Associated Factors, and Outcomes among Intern Physicians in Public Hospitals during the COVID-19 Pandemic: A Cross-Sectional Study

**DOI:** 10.3390/medicina60060962

**Published:** 2024-06-10

**Authors:** Vithawat Surawattanasakul, Wuttipat Kiratipaisarl, Penprapa Siviroj

**Affiliations:** 1 Department of Community Medicine, Faculty of Medicine, Chiang Mai University, Chiang Mai 50200, Thailand; vithawat.surawat@cmu.ac.th (V.S.); wuttipat.k@cmu.ac.th (W.K.); 2 Environmental and Occupational Medicine Excellence Center, Faculty of Medicine, Chiang Mai University, Chiang Mai 50200, Thailand

**Keywords:** presenteeism, manpower shortage, exhaustion, well-being, intern physicians, public hospitals

## Abstract

*Background and Objectives:* Presenteeism, when employees continue to work despite being sick, may have increased among intern physicians during the COVID-19 pandemic due to the necessity of performing unfamiliar tasks. This study aimed to investigate the prevalence of presenteeism among intern physicians (IPs) in Thailand, its associated factors, and outcomes. *Material and Methods:* A total of 254 IPs participated in this cross-sectional study conducted from June to July 2022. Participants completed a nationwide online questionnaire including demographics, financial status, underlying diseases, hospital location and affiliation, department, resource problems, manpower shortage, workload intensity, presenteeism, and its outcomes. IPs were recruited via various social media platforms. Statistical analysis was performed using multivariable zero-inflated Poisson regression and multivariable linear regression. *Results:* The average age of IPs was 25.5 years (SD 1.9), and 57.5% were female. The majority of IPs reported dealing with resource problems (74.8%), insufficient manpower (94.9%), and intense workload (83.5%). Presenteeism was prevalent among 63.8% of IPs, with the most common of the diseases being allergic rhinitis (31.3%). IPs with underlying diseases had an increased rate of presenteeism (adjusted odds ratio (aOR) 2.50, 95% confidence interval (CI) 1.33–4.55). IPs working in community hospitals during their rotations exhibited a lower rate of presenteeism (aOR 0.39, 95% CI 0.16–0.94) compared to other departments within general or regional hospitals. The IPs frequently exposed to insufficient manpower had an increased rate of presenteeism (aOR 4.35, 95% CI 1.02–20.00) compared to those not exposed. Additionally, IPs with presenteeism had more exhaustion (β 1.40, 95% CI 0.33 to 2.46), lower perceived well-being (β −0.65, 95% CI −1.26 to −0.03), and job satisfaction (β −0.33, 95% CI −0.63 to −0.03). *Conclusions:* During COVID-19, intern physicians in Thailand often exhibit presenteeism due to physical conditions, resource scarcity, and personnel shortages, impacting exhaustion, well-being, and job satisfaction. Recommendations include assessing healthcare workforces, allocating resources more effectively, enforcing policies to promote responsible use of sick leave, and implementing sick leave systems.

## 1. Introduction

Presenteeism is when employees persist in working despite illness or other health or productivity issues. It encompasses sickness presenteeism, physical or mental illnesses, and non-sickness personal problems such as stress, personal conflict, fear of job loss, commitment to work, or work culture [1]. The negative consequences of presenteeism extend to both individuals and organizations. Employees may experience increased stress, burnout, and reduced job satisfaction, while organizations may have decreased productivity, lowered employee morale, and increased healthcare costs associated with employees’ health problems [2]. In cases involving physical illnesses, particularly infectious diseases, there is a risk of contagion spreading to coworkers and their patients [3]. Additionally, overwhelmed employees may report to work but exhibit reduced effectiveness [4,5,6,7]. Despite its negative consequences, some perspectives consider presenteeism a form of organizational citizenship, wherein employees demonstrate dedication and commitment to their roles and the organization [1]. However, presenteeism can have effects on both individual health and work quality, particularly in healthcare settings. There are several negative outcomes associated with presenteeism, including poor general health, sleep problems, decreased work performance manifesting as clinical errors, heightened burnout characterized by exhaustion, and exacerbated mental health problems. In healthcare environments, these consequences can directly impact patient care and safety [8,9,10,11].

Evidence indicates that presenteeism is a growing and widespread phenomenon worldwide, with rates ranging from 35% to over 97% among study populations [7]. In 34 European countries, approximately 40% of workers reported experiencing presenteeism at least one day in the previous 12 months [12]. Notably, the prevalence of presenteeism among healthcare professionals, particularly physicians, consistently surpasses the general population. Internationally, physicians exhibit presenteeism rates of approximately 70% to 80% [13,14,15,16,17], a trend also observed among trained physicians [18,19,20]. Similarly, presenteeism among other health professionals, particularly nurses, ranges from 50% to 90% [21,22,23]. In Thailand, 57.9% of resident physicians and 48.1% of healthcare workers reported experiencing presenteeism in the past year [24,25].

Physicians often deliver patient care while ill due to a strong sense of duty and moral obligation toward the welfare of others [26,27,28]. Presenteeism is influenced by several factors, such as the demanding nature of the profession, its high level of specialization, long working hours, physical underlying diseases, and the risk of stress and burnout [1,9,27]. Work-related factors include insufficient resources, concerns about burdening coworkers, apprehension regarding future workload, department affiliation, and position [10,11,13,17,29,30], as well as personal circumstances such as age, sex, and financial constraints contributing to presenteeism among physicians [29]. Additionally, physicians face significant pressures contributing to presenteeism, including heavy workloads, demanding schedules, and challenges in finding adequate substitutes due to manpower shortages [13,23]. They may feel compelled by a strong professional norm against taking sick leave, a sense of responsibility for their patients’ well-being, and a fear of negative consequences for patient care if they take time off due to illness [16].

The COVID-19 pandemic in Thailand resulted in a high infection rate, leading to over two years of limitations and protection for vulnerable populations. This posed unprecedented challenges to health services, which were unprepared for a pandemic of this scale [31,32,33], including a massive influx of COVID-19 patients that exceeded capacity, resulting in overworked staff and resource shortages such as personal protective equipment (PPE), ventilators, and medical supplies in hospitals. Additionally, illness and quarantine measures led to staff shortages, further exacerbating the pressure on the remaining physicians. Healthcare systems had to reorganize hospital wards and units and medical staff and transfer equipment and supplies from other departments to treat COVID-19 patients [34,35]. Therefore, physicians were exposed to extreme workloads and had a high rate of presenteeism during the pandemic. Additionally, Thailand’s labor laws dictate working hours to be 40 to 48 h per week over five days. Employees are entitled to up to 30 days of sick leave per year, with employers paying their full salary [36]. Physicians employed by the Ministry of Public Health have an even more generous allowance of up to 60 working days of sick leave without salary reduction [37]. The COVID-19 pandemic has impacted workplace cultures globally, including in Thailand, where presenteeism was once prevalent [38]. The pandemic has necessitated a shift towards preventing infection spread, emphasizing the importance of sick leave policies and sickness benefits. Under the guidance of the International Labour Organization (ILO), these policies ensure workers receive sufficient income during illness periods [39]. This shift in focus in Thailand reflects a global trend influenced by the pandemic: protecting vulnerable populations and preventing outbreaks.

In Thailand, there is a lack of studies on presenteeism during the COVID-19 pandemic among physicians, particularly interns. The country’s health system is predominantly hospital-based and holds the majority of hospital beds. Medical training in Thailand typically comprises three years of preclinical study followed by three clinical years. Upon completing these stages, medical graduates usually undertake a three-year internship program across various regional, general, or community hospitals [40,41]. Interns are required to work a 10-month rotation in different medical departments within general or regional hospitals, covering specialties such as emergency medicine, surgery, internal medicine, obstetrics and gynecology, and pediatrics during their first year of practice. Additionally, they spend around two months working in community hospitals. Following this, they continue their internships in community hospitals during the second and third years. Passing the first-year internship is vital, as it increases the likelihood of being selected for specialized training in a preferred area. During the COVID-19 pandemic, from October 2021 to September 2022, the total number of outpatients in public hospitals in Thailand totaled 96,806,686 persons or 341,191,247 visits, averaging 3.52 visits per person per year [42]. Consequently, newly graduated physicians face numerous challenges and difficulties as they acclimatize to their new work environment, tasks, and patient care responsibilities. These challenges, combined with high stress levels, may contribute to presenteeism among first-year interns. Therefore, this study aimed to investigate the prevalence of presenteeism and associated factors among intern physicians and explore the association between presenteeism and its consequences as personal outcomes.

## 2. Materials and Methods

### 2.1. Study Design and Participants

This cross-sectional study recruited 254 intern physicians during June and July 2022, which aligned with the COVID-19 pandemic in Thailand. This period corresponds to the final two months of the first year of practice for intern physicians employed by public hospitals, such as regional, general, and community hospitals across Thailand. This study used a nationwide online survey utilizing a web-based online questionnaire. The principal investigator could only access the password-protected database at the webmaster’s request when data curation was carried out. Participants’ birthdays were used as a filter to assist in removing duplicate values. Participant recruitment was conducted through various channels, including social media websites such as Facebook, Twitter, and the Line app, which allows intern physicians to communicate directly. The participants were invited to complete a one-time online survey and the questionnaire on a smartphone, laptop, or computer with internet access through social media. All participants in this study were volunteers, gave their informed consent, and requested that their identities be kept anonymous. Participants received details about the study’s objectives, methods, potential risks and benefits, confidentiality policies, and their rights as research subjects in the consent form. Through the study website, the participants were able to obtain the consent form, which was provided on the first page.

The sample size was calculated using the n4Studies program, which estimated the finite population proportion using the standard formula [43]. This was based on the 2694 total number (N) of Thai intern physicians working in public health hospitals in 2021–2022. The minimum sample size was calculated as 238, assuming a confidence level of 0.095 for the results, an expected proportion (p) of presenteeism among resident physicians in Thailand of 57.9% [25], an absolute precision of 5%, and a maximum error (d) of 0.06. A total of 782 participants accessed the online survey and gave their consent. The survey had broad and easy access for potential respondents. However, only 254 participants were included in the results because they fully completed the survey and met the inclusion criteria of being first-year medical interns. In the final analysis, the study’s sample consisted of 254 respondents, or 32.5% of the total, who accessed the website to complete the survey.

During the preparation of this work, we used ChatGPT 3.5 [44] to check and correct grammatical errors during the manuscript writing process. After using this tool, we reviewed and edited the content as needed, and we take full responsibility for the content of the publication.

### 2.2. Measures

The study employed a self-administered online four-part questionnaire to gather data on participant characteristics, working conditions, presenteeism, and related outcomes. The pilot questionnaire was tested with ten first-year intern physicians at the Maharaj Nakorn Chiang Mai Hospital, Thailand, to improve questions before the study was carried out. However, these interns did not participate in this study.

In Part 1, participant characteristics, participants were asked to provide demographics, health profile, and financial status (5 items in total). Presenteeism-related factors included demographics such as gender and age, underlying medical conditions, both physical and mental, and financial status determined by the perception of three levels of adequacy: inadequate, adequate without savings, and adequate with savings.

Part 2 focused on the working conditions among intern physicians during the first year of internships (6 items in total). These questions addressed work-related information, including the hospital location (north, central, east, west, northeast, and south), hospital departments (emergency medicine, surgery, internal medicine, obstetrics and gynecology, pediatrics, and community hospitals), and hospital affiliation, categorized as Ministry of Public Health (MOPH) and other ministries. Additionally, work-related problems were gathered, including frequency of hospital resource problems and insufficient manpower (responses ranged from infrequent, sometimes, and frequent), as well as perceptions of workload intensity during the first year of internships (categorized as less intense, acceptable intensity, or too intense).

In Part 3, presenteeism was assessed as per a previous study suggesting that self-reported and one-ended, fill-in-blank responses to investigate presenteeism was a practical method of recovering data [27]. We employed a single question (1 item) that asked participants how many days they had engaged in presenteeism during the internship of the previous year: “Have there been any events over the previous 12 months when you have gone to work despite your feeling that you should have taken sick leave due to your health condition?” Responses were divided into two groups: zero indicated no presenteeism and one day or more indicated presenteeism.

In Part 4, the assessment of personal outcomes, we used the Thai version of the following tools among physicians: perceived exhaustion (5 items in total), perceived well-being (3 items), and job satisfaction (4 items). Cronbach’s alpha coefficient for these assessments is shown in Supplementary Table S1.

(1) To assess exhaustion among intern physicians, we asked five questions about their exhaustion, which were from the Maslach Burnout Inventory—Human Services Survey for Medical Personnel, MBI-HSS (MP) translated into Thai and have been used in a previous study [45]. Out of the nine emotional exhaustion (EE) questions, five were selected because these were suitable for the study setting. These questions consisted of “I feel emotionally drained from my work”, “I feel fatigued when I get up in the morning and have to face another day on the job”, “I feel burned out from my work”, “I feel frustrated by my job”, and “I feel like I am at the end of my rope”. Each question was rated on a five-point Likert scale (1 = strongly disagree, 2 = disagree, 3 = neutral, 4 = agree, 5 = strongly agree). Cronbach’s alpha coefficient for these questions was 0.905. For data interpretation, which is derived by adding together the questions’ scores, higher scores indicate more exhaustion.
(2) To assess the perceived well-being and job satisfaction, we employed questions from a previous study on the Quality of Work Life questionnaire on general well-being and job and career aspects among physicians in Thailand [46].

Regarding general well-being, three questions were selected from four items of general well-being in the Quality of Work Life questionnaire, which had a Cronbach’s alpha coefficient of 0.8 [46]. These were deemed appropriate for the study’s setting, and were as follows. “Is your life usually close to ideal?” “Do things generally work well for you?” “Have you been feeling reasonably well lately?” Each question was rated on a five-point Likert scale (1 = strongly disagree, 2 = disagree, 3 = neutral, 4 = agree, 5 = strongly agree). The Cronbach’s alpha coefficient for the questions in this study was 0.722. Higher scores corresponded to greater well-being.

Six questions about job and career satisfaction were included from the Quality of Work Life questionnaire, with a Cronbach’s alpha coefficient of 0.78 [46]. Four of those items were selected for the study’s setting, with a Cronbach’s alpha coefficient of 0.782, as follows. “Do you have the opportunity to apply your abilities at work?” “Are you encouraged to learn new skills?” “Are you satisfied with the career options available to you?” “Are you satisfied with the training provided for your current job?” Each question was rated on a five-point Likert scale (1 = strongly disagree, 2 = disagree, 3 = neutral, 4 = agree, 5 = strongly agree). Higher scores were indicative of higher levels of job satisfaction.

### 2.3. Statistical Analysis

Statistical analysis was performed using STATA software version 16.0 (Stata Corp., College Station, TX, USA). Descriptive analysis determined the characteristics of the sample. Categorical data (participant characteristics and working conditions) are given as frequencies with percentages, and parametric data are described given as means with standard deviation (SD). The chi-squared test and Fisher’s exact test were used to examine statistical differences in the proportion of participants’ characteristics and presenteeism among intern physicians. Multivariable zero-inflated Poisson regression analysis, which is used to solve problems of a dependent variable containing an excess of zero-valued data, was used to calculate the association between the factors and presenteeism with adjusted incident rate ratios (aIRRs) and adjusted ratios (aORs) with 95% confidence intervals (CIs). The results present two models: Poisson count and zero-inflated logit. Additionally, this study examined the association between presenteeism and personal outcomes through regression analyses, including binary and multivariable linear regression. The assumptions for linear regression were assessed and met. Regression coefficients with 95% CIs are reported to represent the magnitude of the observed associations. All statistical tests were two-tailed, and a *p*-value of 0.05 was considered statistically significant. The study’s findings are reported in accordance with the STROBE (Strengthening the Reporting of Observational Studies in Epidemiology) guidelines [47].

### 2.4. Ethical Considerations

This study was conducted following the Declaration of Helsinki guidelines and the protocol was approved by the Research Ethics Committee of the Faculty of Medicine, Chiang Mai University, Thailand (079/2022, date of approval 24 February 2022).

## 3. Results

### 3.1. Participant Characteristics and Working Conditions

Table 1 presents the characteristics of intern physicians and compares those who reported presenteeism with those who reported none. This study collected data through online questionnaires, receiving 254 completed responses. The gender distribution was 57.5% females and 42.5% males, with an average age of 25.5 years (SD 1.9). The majority of participants (64.6%) reported adequate finances with savings, followed by adequate finances without savings (32.3%) and inadequate finances (3.1%). Nearly half of the participants (46.5%) reported underlying diseases, with a significant difference between those with presenteeism (72.0%) and those without (28.0%) (*p* = 0.011). About 18.1% of participants had underlying psychiatric diseases. The majority of interns worked in central, east, and west (42.1%), followed by northeast (29.5%), north (15.0%), and south (13.4%). During this survey, interns worked at various hospital departments, with internal medicine, surgery, community hospitals, and other departments representing 27.6%, 16.9%, 13.8%, and 41.7%, respectively. Most participants were employed by the Ministry of Public Health (85.0%), while 15.0% worked in other government sectors. Most intern physicians reported frequent exposure to hospital resource problems (74.8%), frequent insufficient manpower (94.9%), and their workload being too intense (83.5%). Additionally, significant differences were observed in insufficient manpower among those with presenteeism (65.6%) compared to those without (34.4%) (*p* = 0.011).

### 3.2. Presenteeism in Intern Physicians

Table 2 illustrates the frequency of presenteeism days among intern physicians over the past year. The data indicate that 36.2% of participants reported no presenteeism (92 people). Conversely, the majority of intern physicians experienced presenteeism (162 people, 63.8%). Among those who experienced presenteeism, the distribution of presenteeism days varied, with the most common duration being 3 days (38 people, 14.9%), followed by 2 days (34 people, 13.4%), 5 days (20 people, 7.9%), and 1 day (19 people, 7.5%). Additionally, 7 and 10 days of presenteeism were reported by 5.5% and 6.3% of participants in 30 days.

### 3.3. Physical Underlying Diseases among Intern Physicians

Table 3 shows the frequency of physical underlying diseases of intern physicians categorized by presenteeism and without presenteeism. The most common underlying disease among intern physicians was allergic rhinitis (79 out of 254 people; 31.3%), followed by musculoskeletal disorders (25 people, 9.8%), migraine (10 people, 3.9%), obstructive sleep apnea and skin diseases (equal to 5 people, 2.0%), and gastrointestinal disorders (3 people, 1.2%), respectively. We observed that 60 interns with allergic rhinitis experienced presenteeism, while only 19 reported no presenteeism. Notably, among those experiencing presenteeism, a significantly higher proportion had allergic rhinitis compared to those without presenteeism (*p* = 0.005).

### 3.4. Exploration of the Factors Associated with Presenteeism among Intern Physicians

Table 4 demonstrates the results from the full exploration model analyzed using zero-inflated Poisson regression, which is used for a dependent variable with an excess of zero-valued data, to determine the factors associated with presenteeism among intern physicians. The results present the Poisson count and zero-inflated logit models.

In the Poisson count model, intern physicians who were frequently exposed to resource problems and insufficient manpower in their hospitals showed marginally significantly higher incident rates of presenteeism for durations less than 1 day compared to those who were not exposed. The adjusted incident rate ratios (aIRRs) were 1.42 (95% CI, 0.95 to 2.13, *p* = 0.090) for resource problems and 1.79 (95% CI 0.92–3.46, *p* = 0.085) for insufficient manpower, indicating a 42% and 79% increase in the incident rate of presenteeism for interns, respectively, for interns exposed to these factors.

In the zero-inflated logit model, interns with underlying diseases had a significantly increased rate of having at least one day of presenteeism (aOR 2.50, 95% CI 1.33 to 4.55, *p* = 0.004) compared to those without underlying diseases. Additionally, interns working in community hospitals had a significantly lower rate of having at least one day of presenteeism (aOR 0.39, 95% CI 0.16 to 0.94, *p* = 0.037) than those in other departments or non-major departments. Conversely, intern physicians frequently exposed to insufficient manpower showed a significantly increased rate of having at least one day of presenteeism (aOR 4.35, 95% CI 1.02 to 20.00, *p* = 0.046) compared to those not exposed.

### 3.5. Association between Presenteeism and Personal Outcomes among Intern Physicians

The association between presenteeism and individual outcomes among intern physicians using binary and multivariable linear regression after adjusting for gender and age is shown in Table 5. The results showed that intern physicians with presenteeism had significantly more exhaustion than those without (β 1.40, 95% CI 0.33 to 2.46, *p* = 0.010) (Model A). Conversely, those with presenteeism had lower perceived general well-being (β −0.65, 95% CI −1.26 to −0.03, *p* = 0.041) (Model B) and job satisfaction (β −0.33, 95% CI −0.63 to −0.03, *p* = 0.029) (Model C) than those without. Supplementary Tables S3 and S4 display descriptions of exhaustion, perceptions of general well-being, and job satisfaction among intern physicians by items.

## 4. Discussion

Presenteeism, the phenomenon where physicians are physically present at work but not fully productive due to various reasons such as illness or stress, poses a significant concern for physicians and patient care. This study holds particular significance, as it marks the first study of presenteeism among intern physicians in Thailand. Thai intern physicians have completed six years at medical school and are undergoing supervised clinical practice training in public hospitals for three years. Our findings revealed a prevalence of 63.8% among intern physicians. This is lower than the prevalence of presenteeism among physicians in previous studies from 2009 to 2019 in various countries, including Saudi Arabia, Australia, New Zealand, Italy, Sweden, Norway, Iceland, the USA, and China, ranging from approximately 66% to 86% [13,14,15,16,17,48]. However, the interns involved in this study had higher rates of presenteeism compared to those reported among residents during their training in China, Canada, and the USA. Specifically, the presenteeism rates among residents were 30.7% in China, 59.1% in Canada, and 57.9% in the USA [11,18,19].

Notably, our study conducted during the COVID-19 pandemic indicates that the prevalence of presenteeism among Thai interns may have been influenced by increased patient visits, stress, and workload. This finding is inconsistent with a previous study conducted during the same pandemic in Japan, which found that the prevalence of presenteeism among front-line physicians was lower, at 13.9% [49]. It is essential to note that presenteeism prevalence varies worldwide and may be influenced by various factors, including cultural differences, healthcare system structures, personal characteristics, and work-related factors [50]. Consequently, physicians may internalize this culture, feeling compelled to prioritize their work commitments over their own health needs. This pressure is compounded by the fear of facing criticism from supervisors or receiving negative evaluations if they take time off due to sickness. Furthermore, within hospitals dealing with ongoing challenges, presenteeism can be normalized—where interns feel obligated to be present at work even when unwell—as a necessary response to the prevailing circumstances [9].

Our study revealed that intern physicians with underlying diseases, accounting for 46.5% of the participants, were more likely to report presenteeism than their counterparts without underlying diseases, which is consistent with previous studies [27,51,52,53]. This tendency can be attributed to several reasons, including the pressure to demonstrate their competence and dedication, driven by concerns about potential negative perceptions or consequences if they were to take time off. Interns with underlying diseases may feel a lack of support or understanding from their supervisors or colleagues regarding their health conditions [54,55]. They may also feel a sense of responsibility towards their colleagues and patients in terms of manpower shortages and not wishing to increase the workload of others, which influences their decision to continue working despite being sick [7]. Additionally, there is a fear among these interns regarding the potential adverse impact of taking sick leave on their career progression or future opportunities, leading them to prioritize their work commitments over their own health needs [7]. The most common underlying diseases found in our study were allergic rhinitis and musculoskeletal disorders. A previous study has indicated that participants often explained reasons for presenteeism, such as not feeling sick enough, particularly in cases presumed to be affected by non-infectious disease. It is plausible that due to the chronic nature and tolerable symptoms associated with non-infectious diseases, physicians chose to attend work rather than take sick leave [7].

There is an association between department and presenteeism among intern physicians, particularly when considering the types of hospital they work in, such as community hospitals versus regional or general hospitals. When comparing interns across these hospital types, it becomes evident that presenteeism rates vary. Specifically, interns working in other departments except internal medicine and surgery had higher rates of presenteeism in regional and general hospitals compared to those in community hospitals. Several factors may contribute to this variance: departments within regional or general hospitals in Thailand, such as emergency medicine, surgery, internal medicine, obstetrics and gynecology, and pediatrics, typically face higher patient volumes, more acute cases, and greater work demands compared to community hospitals [40], and often experience heightened pressure to maintain staffing levels and effectively manage patient care needs [56]. Additionally, the culture within regional or general hospitals tends to prioritize dedication to patient care and fosters a sense of team responsibility, thereby amplifying physicians’ sense of obligation to colleagues and patients [8]. However, the doctor-to-patient ratio in community hospitals is lower than in regional or general hospitals. Predominantly, general practitioners make up the majority of the medical staff in community hospitals. The workload is lighter than in larger hospitals, and there are fewer patients overall. Proximity facilitates easier communication among interns and medical staff, enabling them to request time off when sick and seek assistance from colleagues to manage the workload.

Our study demonstrates that intern physicians who were frequently exposed to resource problems and insufficient manpower in their hospitals exhibit significantly higher rates of presenteeism, aligning with findings from previous studies [27,28]. Interns working in hospitals facing these challenges often find themselves experiencing heavier workloads and increased responsibilities. With fewer staff members available to cover shifts or assist with patient care, interns may feel compelled to work longer hours and take on additional tasks [9,16,28]. These resource-constrained hospitals often lack essential support services such as nursing assistance, administrative support, or access to specialized equipment, making it difficult to meet patient care demands effectively [54]. Interns working in environments characterized by resource constraints and manpower shortages often experience a heightened sense of responsibility to provide adequate care to patients despite these limitations.

In our study, we found that several factors, including socio-demographics (age and financial status), hospital location and affiliation, and an intense workload, showed no association with presenteeism among intern physicians, which is consistent with previous studies [14,29,54]. However, gender factors in previous studies have been associated with presenteeism among physicians [14,53,54,57]. This lack of association may be attributed to the relative homogeneity of interns, particularly regarding socio-demographic characteristics. When there is limited variability within these factors, detecting meaningful associations becomes challenging. Moreover, the timing of the study could have influenced the results. For example, perceptions of workload may vary over time, and the timing of data collection might impact the observed associations. It seems that presenteeism is influenced more significantly by factors such as work-related factors, professional culture, organizational behaviors, or individual health status than personal factors. Additionally, we observed that underlying psychiatric diseases among intern physicians were not associated with presenteeism, which is inconsistent with a previous study [54]. Individuals with underlying psychiatric diseases may be inclined to conceal their conditions due to stigma or fear of discrimination [58]. Moreover, the measurement of underlying psychiatric diseases within this study may have been limited in accuracy or reliability, possibly due to issues such as self-reporting bias or imprecise diagnostic criteria. Consequently, these interns may be less likely to report presenteeism related to their mental health problems. It is worth noting that the dynamics of presenteeism and its associations with various factors can vary across different populations and settings.

Our study revealed a significant association between presenteeism and personal outcomes such as exhaustion, perceived general well-being, and job satisfaction. Several factors contribute to the association: interns often experience pressure due to heavy workloads and long work hours, particularly exacerbated during the COVID-19 pandemic. In Thailand, newly graduated intern physicians face high pressure to deliver frontline patient care. Throughout their twelve-month internship, they rotate through different hospital departments [40,41], adding to the complexity of workplace dynamics, clinical pressure, and patient care responsibilities—significant stressors in academic settings. Furthermore, the fear of negative evaluations or criticism from supervisors for taking sick leave further intensifies interns’ stress levels and contributes to their exhaustion and reduced well-being and job satisfaction [9,59]. This suggests that job satisfaction is influenced by a multitude of factors beyond presenteeism. Workplace dynamics, individual perceptions, and organizational culture within hospitals all play a role in shaping interns’ satisfaction with their jobs. Hospital managers should consider these complex factors when addressing issues related to interns’ well-being and job satisfaction [60].

This study offers valuable insights into the presenteeism among intern physicians in Thailand, potentially informing improvements in training programs to enhance physician health outcomes. However, several limitations should be acknowledged. Firstly, its cross-sectional design and reliance on self-reported data may have led to inflated assumptions about association and causal relationships. Future research would benefit from incorporating multiple data sources, including medical records, to minimize methodological biases. Secondly, the 32.5% response rate, which is less than 10% of the first-year intern physician population, and using a convenience sampling method raise concerns about selection bias and the generalizability of findings to all. While efforts were made to compare the study sample with the broader population, caution is needed when extrapolating results. Thirdly, potential confounding factors influencing presenteeism, such as workplace stressors, fatigue, burnout, and other mental health issues, as well as leadership styles and organizational culture [54,61,62,63,64], may not have been accounted for in this study. Presenteeism in physicians requires a multifaceted approach that addresses systemic issues related to workplace culture, workload management, and support systems while promoting physician well-being and self-care practices [50,65]. Further research should aim to overcome these limitations through longitudinal studies with diverse data sources and robust sampling techniques to enhance validity and generalizability.

## 5. Conclusions

The prevalence of presenteeism, indicating working despite being sick, was notably high at 63.8% among intern physicians in Thailand. Several factors contributed to this increased prevalence among Thai intern physicians, including physical underlying disease, working in regional or general hospitals, challenges related to resource shortage, and insufficient manpower. Additionally, there are associations between presenteeism and its consequences, such as exhaustion, general well-being, and job satisfaction, among intern physicians. It is crucial to assess the available healthcare workforce and allocate it appropriately based on the hospital’s specific context. Implementing a system that enables individuals to take sick leave without feeling guilty, along with providing suitable replacements, is a key strategy for reducing presenteeism among intern physicians. In light of these findings, healthcare institutions should develop and enforce policies that actively discourage presenteeism while promoting responsible utilization of sick leave entitlements, and regularly detecting mental health problems, well-being, and job satisfaction, especially among physicians with presenteeism.

Further research should explore factors contributing to presenteeism, including cultural differences, healthcare systems, psychiatric health problems, and the specific characteristics of the study participants. For developing healthcare service policies, the impact of presenteeism on physicians’ health, quality of work life, and patient care should be considered. Understanding these factors’ interplay can inform targeted interventions to address presenteeism effectively. Interventions aimed at fostering a healthier work environment should be carefully designed and implemented, such as tailored support for physicians with physical underlying diseases, flexible work arrangements, and effective symptom management strategies. These interventions can create a more productive and supportive work environment that aligns with the professional duties of physicians.

## Figures and Tables

**Table 1:** Socio-demographic and health characteristics and working conditions of intern physicians.

Determinants	Total (*n* = 254)	*n* (%)		*p*-Value
		No Presenteeism (*n* = 92)	Presenteeism ^≠^ (*n* = 162)	
Gender				
Male	108 (42.5)	46 (42.6)	62 (57.4)	0.069
Female	146 (57.5)	46 (31.5)	100 (68.5)	
Age (years), mean (SD)	25.5 (1.9)	25.6 (1.7)	25.5 (2.0)	0.700
Perceived financial status				
Inadequate	8 (3.1)	4 (50.0)	4 (50.0)	0.231
Adequate without savings	82 (32.3)	24 (29.3)	58 (70.7)	
Adequate with savings	164 (64.6)	64 (39.0)	100 (61.0)	
Physical underlying diseases	118 (46.5)	33 (28.0)	85 (72.0)	0.011 *
Psychiatric underlying diseases	46 (18.1)	18 (39.1)	28 (60.9)	0.650
Region of hospital location				
Central/east/west	107 (42.1)	35 (32.7)	72 (67.3)	0.609
North	38 (15.0)	17 (44.7)	21 (55.3)	
Northeast	75 (29.5)	27 (36.0)	48 (64.0)	
South	34 (13.4)	13 (38.2)	21 (61.8)	
Department				
Internal medicine	70 (27.6)	25 (35.7)	45 (64.3)	0.237
Surgery	43 (16.9)	14 (32.6)	29 (67.4)	
Community hospitals	35 (13.8)	18 (51.4)	17 (48.6)	
Others	106 (41.7)	35 (33.0)	71 (67.0)	
Hospital affiliation				
Ministry of Public Health	216 (85.0)	76 (35.2)	140 (64.8)	0.413
Other ministries	38 (15.0)	16 (42.1)	22 (57.9)	
Hospital resource problems				
Infrequent to sometimes	64 (25.2)	25 (39.1)	39 (60.9)	0.584
Frequent	190 (74.8)	67 (72.8)	123 (64.7)	
Insufficient manpower				
Infrequent to sometimes	13 (5.1)	9 (69.2)	4 (30.8)	0.011*
Frequent	241 (94.9)	83 (34.4)	158 (65.6)	
Intense workload				
Acceptable intensity	42 (16.5)	20 (47.6)	22 (52.4)	0.093
Too intense	212 (83.5)	72 (34.0)	140 (66.0)	

^≠^ Presenteeism is defined as working while sick for one or more days in the past year; statistical analysis with chi-squared test; * significant association at 0.05. Abbreviation: SD, standard deviation.

**Table 2:** Frequency and percentage of presentation days among intern physicians.

	Number of Presenteeism Days (*n* = 254)												
	0	1	2	3	4	5	7	10	12	13	15	20	30
*n*	92	19	34	38	8	20	14	16	2	1	4	3	3
%	36.2	7.5	13.4	14.9	3.1	7.9	5.5	6.3	0.8	0.4	1.6	1.2	1.2

**Table 3:** Underlying diseases of intern physicians with presenteeism and without.

Underlying Diseases	*n* (%)			*p*-Value
	Total (*n* = 254)	No Presenteeism (*n* = 92)	Presenteeism ^≠^ (*n* = 162)	
Allergic rhinitis	79 (31.3)	19 (20.9)	60 (37.3)	0.005 ^a,^*
Musculoskeletal disorders	25 (9.8)	9 (9.7)	16 (9.9)	1.00 ^a^
Migraine	10 (3.9)	1 (1.1)	9 (5.6)	0.098 ^b^
Obstructive sleep apnea	5 (2.0)	2 (2.2)	3 (1.9)	1.00 ^b^
Skin diseases (e.g., atopic dermatitis and chronic urticaria)	5 (2.0)	1 (1.1)	4 (2.5)	0.66 ^b^
Gastrointestinal disorders (e.g., GERD, IBS, and dyspepsia)	3 (1.2)	0 (0.0)	3 (1.9)	0.30 ^b^
Others	17 (6.7)	6 (6.5)	11 (6.8)	1.00 ^a^

^≠^ Presenteeism is defined as working while sick for one or more days in the past year; ^a^ statistical analysis with chi-squared test, ^b^ statistical analysis with Fisher’s exact test. Gastrointestinal diseases: GERD, gastro-esophageal reflux disease (1 person), IBS, irritable bowel syndrome (1 person), dyspepsia (1 person); others: asthma (3 people), endometriosis (2 people), hypertension (1 person), malignancy (1 person), thalassemia trait (1 person), not specified (7 people). * Significant association at 0.01.

**Table 4:** Multivariable zero-inflated Poisson regression showing factors associated with adjusted presenteeism incident rate ratio and adjusted odds ratio for having at least one day of presenteeism among intern physicians.

Determinants (*n* = 254)	Poisson Count Model			Zero-Inflated Logit Model		
	aIRR	95% CI	*p*-Value	aOR	95% CI	*p*-Value
Gender						
Male	0.98	(0.69, 1.39)	0.917	0.62	(0.34, 1.10)	0.103
Female	1			1		
Age (each 1-year increase)	0.95	(0.88, 1.02)	0.133	1.03	(0.86, 1.22)	0.769
Perceived financial status						
Inadequate	1.60	(0.61, 4.16)	0.337	0.34	(0.07, 1.72)	0.192
Adequate without savings	1			1		
Adequate with savings	1.00	(0.70, 1.43)	0.994	0.62	(0.31, 1.22)	0.167
Physical underlying diseases						
No	1			1		
Yes	1.10	(0.78, 1.55)	0.603	2.50	(1.33, 4.55)	0.004 **
Psychiatric underlying diseases						
No	1			1		
Yes	1.04	(0.71, 1.51)	0.849	0.66	(0.30, 1.45)	0.298
Region of hospital location						
Central/east/west	1			1		
North	0.71	(0.41, 1.21)	0.209	0.54	(0.23, 1.28)	0.161
Northeast	0.84	(0.56, 1.28)	0.423	0.81	(0.40, 1.67)	0.571
South	1.55	(0.90, 2.67)	0.117	0.75	(0.30, 1.85)	0.539
Department affiliation						
Others	1			1		
Internal medicine	1.09	(0.77, 1.54)	0.629	0.68	(0.34, 1.37)	0.284
Surgery	1.27	(0.81, 1.97)	0.296	0.88	(0.36, 2.17)	0.797
Community hospitals	1.00	(0.58, 1.73)	0.992	0.39	(0.16, 0.94)	0.037 *
Hospital affiliation						
Ministry of Public Health	1.07	(0.60, 1.94)	0.812	1.32	(0.52, 3.33)	0.563
Other ministries	1					
Hospital resource problems						
Infrequent to sometimes	1			1		
Frequent	1.42	(0.95, 2.13)	0.090	0.84	(0.42, 1.69)	0.632
Insufficient manpower						
Infrequent to sometimes	1			1		
Frequent	1.79	(0.92, 3.46)	0.085	4.35	(1.02, 20.00)	0.046 *
Intense workload						
Acceptable intensity	1			1		
Too intense	0.82	(0.47, 1.43)	0.476	1.59	(0.70, 3.57)	0.267

Statistical analysis using multivariable zero-inflated Poisson regression; * significant association at 0.05, ** significant association at 0.01. Abbreviations: aIRR, adjusted incident rate ratio; aOR, adjusted odds ratio; CI, confidence interval.

**Table 5:** The association between presenteeism and personal outcomes among intern physicians.

Model	Personal Outcomes (*n* = 254)	Presenteeism, Mean ± SD		β Coef. ^a^ (SE)	95% CI	*p*-Value	β Coef. ^b^ (SE)	95% CI	*p*-Value
		No	Yes						
A	Exhaustion (scores) (*n* = 254)	12.96 ±4.82	14.53 ±3.73	1.57 (0.54)	0.51 to 2.64	0.004 **	1.40 (0.54)	0.33 to 2.46	0.010 **
B	Perceived general well-being (scores) (*n* = 224)	8.17 ±2.23	7.48 ±2.21	−0.69 (0.31)	−1.31 to −0.07	0.028 *	−0.65 (0.31)	−1.26 to −0.03	0.041 *
C	Job satisfaction (scores) (*n* = 224)	9.34 ±2.03	9.423 ±2.51	−0.34 (0.15)	−0.64 to −0.05	0.024 *	−0.33 (0.15)	−0.63 to −0.03	0.029 *

Model A, the association between presenteeism and exhaustion; Model B, the association between presenteeism and perceived general well-being; Model C, the association between presenteeism and job satisfaction; statistical analysis using ^a^ bivariate linear regression and **^b^** multivariable linear regression adjusted for gender and age; reference group is no presenteeism. * Significant association at 0.05, ** Significant association at 0.01. Abbreviations: SD, standard deviation; β Coef., standardized beta coefficient; SE, standard error; CI, confidence interval.

## Data Availability

The data presented in this study are available from the corresponding author on reasonable request.

## Supplementary Materials

The following supporting information can be downloaded at https://www.mdpi.com/article/10.3390/medicina60060962/s1. Table S1: Reliability assessment of exhaustion, perception of general well-being, and job satisfaction questionnaires; Table S2: Frequency and percentage of exhaustion questions among intern physicians with presenteeism and without; Table S3: Frequency and percentage of perception of general well-being, and job satisfaction questions among intern physicians with presenteeism and without.
