# Supplementary material for: Association between Presenteeism, Associated Factors, and Outcomes among Intern Physicians in Public Hospitals during the COVID-19 Pandemic: A Cross-Sectional Study
Source: Medicina (Kaunas). 2024 Jun 10;60(6):962. doi: 10.3390/medicina60060962 (PMC11205852; doi:10.3390/medicina60060962)
Supplement: Supplementary file 1 [file medicina-60-00962-s001.zip › medicina-3035777-supplementary/240606 Supplementary Table S1.pdf]

Table S1: The reliability assessment of exhaustion, perception of general well-being, and job satisfaction questionnaires.

Questions	Sign	Mean	SD	Corrected Item-Total Correlation	Cronbach's Alpha if Item Deleted
Exhaustion					
1. I feel emotionally drained from my work.	-	4.536	1.533	0.783	0.881
2. I feel fatigued when I get up in the morning and have to face another day on the job.	-	4.672	1.622	0.737	0.889
3. I feel burned out from my work	-	4.425	1.679	0.838	0.867
4. I feel frustrated by my job.	-	4.092	1.739	0.746	0.887
5. I feel like I am at the end of my rope.	-	3.534	1.898	0.723	0.895
**Test scale (Cronbach's alpha coefficient 5 items)**				**0.905**	
Perception of general well-being					
1. Is your life usually close to ideal?	+	1.987	0.868	0.477	0.709
2. Do things generally work well for you?	+	2.858	0.905	0.649	0.504
3. Have you been feeling reasonably well currently?	+	2.862	1.006	0.516	0.674
**Test scale (Cronbach's alpha coefficient 3 items)**				**0.722**	
Job satisfaction					
1. Do you have the opportunity to apply your abilities at work?	+	3.489	0.917	0.460	0.787
2. Are you encouraged to learn new skills?	+	2.978	1.006	0.554	0.745
3. Are you satisfied with the career options available to you?	+	2.929	1.079	0.672	0.682
4. Are you satisfied with the training provided for your current job?	+	3.080	1.091	0.671	0.682
**Test scale (Cronbach's alpha coefficient 4 items)**				**0.782**	
