# Supplementary material for: Association between Presenteeism, Associated Factors, and Outcomes among Intern Physicians in Public Hospitals during the COVID-19 Pandemic: A Cross-Sectional Study
Source: Medicina (Kaunas). 2024 Jun 10;60(6):962. doi: 10.3390/medicina60060962 (PMC11205852; doi:10.3390/medicina60060962)
Supplement: Supplementary file 1 [file medicina-60-00962-s001.zip › medicina-3035777-supplementary/240606 Supplementary Table S2.pdf]

Table S2. Frequency and percentage of exhaustion questions among intern physicians with presenteeism and without.

**Questions (*n* = 254)**	***n* (%)**														***P*-value**
	**No presenteeism (*n* = 92)**							**Presenteeism (*n* = 162)**							
	**0**	**1**	**2**	**3**	**4**	**5**	**6**	**0**	**1**	**2**	**3**	**4**	**5**	**6**	
Exhaustion															
1. I feel emotionally drained from my work.	2 (2.2)	6 (6.5)	7 (7.6)	10 (10.9)	9 (9.8)	36 (39.1)	22 (23.9)	1 (0.6)	4 (2.5)	7 (4.3)	13 (8.0)	22 (13.6)	50 (30.9)	65 (40.1)	0.053
2. I feel fatigued when I get up in the morning and have to face another day on the job.	4 (4.3)	7 (7.6)	4 (4.3)	6 (6.5)	13 (14.1)	30 (32.6)	28 (30.4)	4 (2.5)	2 (1.2)	4 (2.5)	10 (6.2)	15 (9.3)	42 (25.9)	85 (52.5)	0.008 *
3. I feel burned out from my work	5 (5.4)	8 (8.7)	4 (4.3)	8 (8.7)	12 (13.0)	27 (29.3)	28 (30.4)	5 (3.1)	5 (3.1)	4 (2.5)	21 (13.0)	24 (14.8)	41 (25.3)	62 (38.3)	0.272
4. I feel frustrated by my job.	6 (6.5)	6 (6.5)	11 (12.0)	11 (12.0)	10 (10.9)	28 (30.4)	20 (21.7)	8 (4.9)	5 (3.1)	11 (6.8)	25 (15.4)	25 (15.4)	42 (25.9)	46 (69.7)	0.368
5. I feel like I am at the end of my rope.	8 (8.7)	14 (15.2)	14 (15.2)	10 (10.9)	20 (21.7)	17 (18.5)	9 (9.8)	14 (8.6)	14 (8.6)	12 (7.4)	22 (13.6)	27 (16.7)	38 (23.5)	35 (21.6)	0.052

≠ Presenteeism is defined as working while sick for one or more days in the past year; Sevene-point Likert scale: 0 = never, 1 = 2–3 times a year, 2 = once a month, 3 = 2–3 times a month, 4 = once a week, 5 = 2–3 times a week, 6 = every day; Statistical analysis with Fisher's Exact test; * Significant association at 0.05.
