# Supplementary material for: Association between Presenteeism, Associated Factors, and Outcomes among Intern Physicians in Public Hospitals during the COVID-19 Pandemic: A Cross-Sectional Study
Source: Medicina (Kaunas). 2024 Jun 10;60(6):962. doi: 10.3390/medicina60060962 (PMC11205852; doi:10.3390/medicina60060962)
Supplement: Supplementary file 1 [file medicina-60-00962-s001.zip › medicina-3035777-supplementary/240606 Supplementary Table S3.pdf]

Table S3. Frequency and percentage of perception of general well-being, and job satisfaction questions among intern physicians with presenteeism and without.

**Questions (*n* = 224)**	**Response of *n* (%)**										***P*-value**
	**No presenteeism (*n* = 76)**					**Presenteeism (*n* = 148)**					
	**1**	**2**	**3**	**4**	**5**	**1**	**2**	**3**	**4**	**5**	
Perception of general well-being											
1. Is your life usually close to ideal?	0 (0.0)	21 (27.6)	30 (39.5)	23 (30.3)	2 (20.6)	0 (0.0)	55 (37.2)	55 (37.2)	30 (20.3)	8 (5.4)	0.237
2. Do things generally work well for you?	6 (7.9)	13 (17.1)	31 (40.8)	25 (32.9)	1 (1.3)	14 (9.5)	33 (22.3)	75 (50.7)	24 (16.2)	2 (1.4)	0.078
3. Have you been feeling reasonably well currently?	9 (11.8)	9 (11.8)	28 (36.8)	28 (36.8)	2 (2.6)	24 (16.2)	18 (12.2)	77 (52.0)	27 (18.2)	2 (1.4)	0.027*
Job satisfaction											
1. Do you have the opportunity to apply your abilities at work?	2 (2.6)	9 (11.8)	30 (39.5)	26 (34.2)	9 (11.8)	5 (3.4)	12 (8.1)	45 (30.4)	71 (48.0)	15 (10.1)	0.336
2. Are you encouraged to learn new skills?	8 (10.5)	14 (18.4)	35 (46.1)	17 (22.4)	2 2.6	13 (8.8)	30 (20.3)	52 (35.1)	46 (31.1)	7 (4.7)	0.466
3. Are you satisfied with the career options available to you?	7 (9.2)	14 (18.4)	28 (36.8)	22 (28.9)	5 (6.6)	23 (15.5)	23 (15.5)	60 (40.5)	35 (23.6)	7 (4.7)	0.578
4. Are you satisfied with the training provided for your current job?	10 (13.2)	9 (11.8)	26 (34.2)	25 (32.9)	6 (7.9)	17 (11.5)	19 (12.8)	59 (39.9)	43 (29.1)	10 (6.8)	0.917

≠ Presenteeism is defined as working while sick for one or more days in the past year; Five-point Likert scale: 1 = strongly disagree, 2 = disagree, 3 = neutral, 4 = agree, 5 = strongly agree; Statistical analysis with Fisher's exact test; * Significant association at 0.05.
